# Factors Associated with Sarcopenia Among Vietnamese Elderly Outpatients with Chronic Musculoskeletal Disorders: A Cross-Sectional Study

**DOI:** 10.3390/jcm15135138

**Published:** 2026-07-01

**Authors:** Nguyen The Diep, Tien Van Nguyen, Nguyen Trong Duynh

**Affiliations:** 1 Department of Traumatology, Thai Binh University of Medicine and Pharmacy, Hung Yen 17000, Vietnam; diepnguyentheytb@gmail.com; 2 Department of Healthcare Organisation and Management, Faculty of Public Health, Thai Binh University of Medicine and Pharmacy, Hung Yen 17000, Vietnam; 3 Department of Adult and Geriatric Nursing, Faculty of Nursing, Thai Binh University of Medicine and Pharmacy, Hung Yen 17000, Vietnam; duynhtbmu2010@gmail.com

**Keywords:** Asian Working Group for Sarcopenia, coexisting knee osteoarthritis, chronic spinal pain, secondary analysis, fall history, sleep quality, Vietnam

## Abstract

**Background/Objectives**: Sarcopenia may compound mobility limitations and fall vulnerability among older adults with coexisting knee osteoarthritis (KOA) and chronic spinal pain. This secondary analysis of a previously reported or substantially overlapping cohort estimated the proportion meeting Asian Working Group for Sarcopenia (AWGS) 2019 criteria and explored additional adjusted associations in a selected Vietnamese outpatient sample. **Methods**: A hospital-based secondary cross-sectional analysis included 88 outpatients aged ≥ 60 years (mean age, 70.5 ± 6.7 years; 69 women, 78.4%) with coexisting KOA and chronic spinal pain who were recruited by convenience sampling at Thai Binh General Hospital from May to October 2024. Source-record verification confirmed that all of the analytic participants had both diagnoses. Their muscle mass, grip strength, and gait speed were assessed using the InBody 770, an InGrip handgrip dynamometer, and a 15-foot walk test, respectively. The prespecified primary classification used AWGS 2019. The AWGS 2025 framework was considered during revision, but numerical reclassification was not feasible because the retained participant-level analytic dataset contained the derived AWGS 2019 outcome and covariates used in the reported regression and CHAID analyses, but not the original continuous age, appendicular skeletal muscle mass index, handgrip values, or a complete raw component record sufficient to independently reconstruct the AWGS 2019 status or apply AWGS 2025 thresholds. Multivariable logistic regression and CHAID were treated as exploratory. **Results**: Under AWGS 2019, 36/88 participants (40.9%) had sarcopenia, including 15 (17.0%) with severe sarcopenia. All 88 participants had both KOA and chronic spinal pain; therefore, diagnostic-category subgroup comparisons were not applicable. In the exploratory adjusted analysis, an age > 70 years (adjusted odds ratio [AOR]: 9.00, 95% confidence interval [CI]: 2.40–33.60), a history of falls (AOR: 6.33, 95% CI: 2.77–14.45), low educational attainment (AOR: 2.86, 95% CI: 1.46–5.61), and a higher Pittsburgh Sleep Quality Index score (AOR: 1.16, 95% CI: 1.02–1.32) remained associated with sarcopenia. Wide CIs and approximately 4.5 events per regression coefficient indicated substantial imprecision. **Conclusions**: This secondary report provides setting-specific descriptive evidence rather than independent replication, a validated prediction tool, or a fully auditable reconstruction of the original AWGS component measurements. Because AWGS 2025 reclassification could not be reconstructed from the retained dataset and raw component records, the AWGS 2019 estimate should not be treated as directly interchangeable with the estimates generated under the updated framework. The observed associations and within-sample subgroup patterns require confirmation in larger, prospectively auditable studies.

## 1. Introduction

Population aging has increased the clinical relevance of sarcopenia, a syndrome characterized by the loss of skeletal muscle mass and strength that is associated with functional decline, falls, frailty, and mortality [1,2,3]. In Vietnam, early recognition is relevant to both individual care and service planning because preventable disability and fall-related complications may increase the rehabilitation needs, caregiver burden, and healthcare use.

Older adults with coexisting knee osteoarthritis (KOA) and chronic spinal pain may be particularly vulnerable because pain, reduced mobility, and activity avoidance can contribute to deconditioning and muscle dysfunction [4,5]. Musculoskeletal outpatient clinics may therefore offer a practical setting for identifying patients who warrant a formal muscle assessment. However, the available Vietnamese literature primarily involves community-dwelling adults or geriatric-clinic populations rather than patients selected through musculoskeletal services [6,7,8,9]. The knowledge gap addressed by this study is deliberately narrow. First, local data using standardized AWGS criteria are limited for selected older Vietnamese musculoskeletal outpatients with coexisting KOA and chronic spinal pain. Second, because single-condition and combined-condition musculoskeletal samples have different implications for target-population interpretation, the diagnostic composition of the retained analytic sample required explicit source-record verification. Third, it remains uncertain whether readily obtainable clinical and social characteristics—including the fall history, educational attainment, sleep quality, and pain severity—show exploratory adjusted associations that could inform future case-finding research. The manuscript does not propose a new predictive model or definitive risk-stratification tool.

A related publication from the same research program reported an 88-participant cohort with the same or a substantially overlapping diagnostic profile and identical core AWGS 2019 findings, including the 40.9% sarcopenia proportion and the age > 70 years association [10]. The available documentation indicates that the samples are the same or substantially overlapping; however, a participant-level linkage key was not retained, so exact person-by-person overlap cannot be reconstructed. For this reason, the present manuscript is framed as a secondary exploratory analysis of a previously reported cohort rather than as an independent cohort study or independent replication. Its incremental contribution is limited to the diagnostic-composition clarification, additional routinely collected variables, exploratory adjusted associations, a descriptive CHAID display, and interpretation under the AWGS 2025 framework. The findings shared with the earlier report are explicitly identified, as previously reported.

The AWGS 2025 consensus update shifted the emphasis from a disease-only framework to life-course muscle health, expanded diagnostic thresholds to adults aged 50–64 years, simplified diagnoses to the concurrent presence of low muscle mass and low muscle strength, and treated physical performance as an outcome measure rather than a diagnostic requirement [11]. Because data collection and the prespecified primary analysis preceded this update, AWGS 2019 remains the primary framework. AWGS 2019-based estimates should not be assumed to be directly comparable with AWGS 2025 estimates. A participant-level sensitivity reclassification was considered; however, the retained de-identified participant-level file contained the derived AWGS 2019 status and covariates used in the secondary regression and CHAID analyses, but not the original continuous age, ASMI, handgrip values, or complete raw component records needed to reconstruct the AWGS 2019 status independently or to apply the updated age-specific thresholds. In particular, the retained 60–65-year age category could not distinguish participants aged 60–64 years from those aged 65 years.

Logistic regression was used to estimate exploratory adjusted associations. A CHAID tree was retained only as a descriptive partitioning display of within-sample patterns. Given the modest single-center sample, neither analysis was intended to establish the predictive performance, a clinical decision rule, or causal effects.

Accordingly, this secondary cross-sectional analysis of a previously reported or substantially overlapping cohort aimed to (1) describe the AWGS 2019 sarcopenia proportion in the verified combined-diagnosis sample; (2) report the diagnostic composition after source-record verification and determine whether single-condition comparisons were possible; (3) interpret the AWGS 2019 estimate in light of AWGS 2025 and specify which retained data do and do not support reclassification; and (4) explore additional adjusted associations and within-sample CHAID patterns. All inferential and subgroup analyses were interpreted as hypothesis-generating, and the report was not presented as independent replication or as a fully auditable reconstruction of the original AWGS component data.

## 2. Materials and Methods

The study was conducted in accordance with the Declaration of Helsinki. The protocol received scientific approval from Thai Binh University of Medicine and Pharmacy (decision No. 855, dated 2 May 2024) and ethical approval from the Ethics Council of Thai Binh University of Medicine and Pharmacy (decision No. 0224/IRB, dated 22 July 2024); the Board of Directors of Thai Binh General Hospital authorized study conduct. Written informed consent was obtained from all the participants before data collection.

### 2.1. Study Design, Setting, and Relationship to the Related Publication

This secondary cross-sectional analysis used data collected from May to October 2024 in the Musculoskeletal Department at Thai Binh General Hospital, Vietnam. The core AWGS 2019 prevalence, frailty results, and age association from an 88-participant cohort with the same or a substantially overlapping sample were previously reported in a related article [10]. The present manuscript is therefore not presented as an independent cohort study or independent replication; it evaluates additional routinely collected variables, exploratory adjusted associations, a descriptive CHAID display, and the implications of the AWGS 2025 update conditional on the retained derived AWGS 2019 outcome. The reporting was reviewed against the STROBE recommendations for cross-sectional studies. Because the participants were recruited from a selected outpatient clinic and all analytic participants had coexisting KOA and chronic spinal pain, the findings are not intended to represent community-dwelling older adults, broader geriatric populations, or patients with isolated KOA or isolated chronic spinal pain.

### 2.2. Participants and Sampling

The study included older outpatients who met the following verified criteria: (1) an age ≥ 60 years; (2) a diagnosis of both KOA, according to the American College of Rheumatology criteria, and chronic spinal pain; and (3) a willingness to participate. The exclusion criteria were severe cognitive impairment, acute illness, a severe functional limitation preventing performance testing, neurological disorders likely to substantially affect gait or handgrip testing, maintenance dialysis, implanted electrical devices, limb amputation, marked fluid retention, or other contraindications to body-composition assessments. Eligible participants were enrolled by convenience sampling during routine outpatient visits from May to October 2024. The final analytic sample comprised 88 participants and corresponded to the same or a substantially overlapping sample described in the related report [10]. Source-record verification for this secondary report was performed by reviewing the retained participant list, diagnostic fields in the study data-collection file, and the diagnostic profile described in the related publication. This verification confirmed that every retained analytic participant had both conditions; no KOA-only or chronic-spinal-pain-only participants were present in the final analytic dataset. The resulting diagnostic composition is reported in Section 3.2.

A participant-flow summary was prepared. The retained study file did not include a complete screening log for all potentially eligible outpatient visits or refusals; therefore, these totals could not be reconstructed. The traceable stages from consent through the final analysis are reported without estimating or fabricating unavailable counts.

The minimum sample size was estimated using the single-population proportion formula:

$$n=Z_{\left(1-\alpha /2\right)}^{2}\times p(1-p)/d^{2}$$

where Z = 1.96 for a two-sided 95% confidence level, p is the expected sarcopenia prevalence, and d is the desired absolute precision. We used p = 0.648 from a Vietnamese older-adult study [6] and d = 0.10, yielding a minimum sample size of 88. This calculation was intended only for a prevalence description and did not provide adequate power justification for multivariable inference or predictive modeling. The final model included 36 sarcopenia events and eight regression coefficients (approximately 4.5 events per coefficient), increasing the risk of sparse-data bias, unstable estimates, and wide confidence intervals.

### 2.3. Data Collection and Measurements

Data were collected through structured interviews and standardized clinical assessments by trained study staff using a prespecified data-collection form.

The demographic and clinical variables included the age, sex, education level, occupation, living situation, body mass index, smoking, alcohol use, history of falls in the previous 12 months, sleep quality assessed using the Pittsburgh Sleep Quality Index (PSQI; range 0–21, with higher scores indicating poorer sleep quality), comorbidities, severity of knee osteoarthritis, chronic spinal pain severity, number of medications used, and frailty status assessed using the Fried phenotype criteria. Participants meeting three or more Fried criteria were classified as frail. Frailty was summarized descriptively and by coexistence with sarcopenia. It was not included in the primary multivariable model because only eight participants were frail, and the Fried phenotype includes components that overlap with sarcopenia assessments, particularly weakness and slowness. Including frailty in the same small regression model could therefore introduce sparse-data problems and potential overadjustment. Nevertheless, frailty was recognized as an overlapping, but distinct, geriatric construct that may act as a confounder, mediator, or coexisting syndrome.

The habitual physical activity, dietary intake, protein consumption, malnutrition risk, rehabilitation exposure, and inflammatory biomarkers were not measured because these items were not included in the original outpatient data-collection form. This omission was considered a major source of residual confounding when interpreting the adjusted associations.

Primary sarcopenia classification (AWGS 2019): During the original analysis, AWGS 2019 classifications were generated from participant-level measurements of the muscle mass, handgrip strength, and gait speed available to the study team at that time. A low muscle mass was defined as an appendicular skeletal muscle mass index (ASMI) < 7.0 kg/m^2^ for men and <5.7 kg/m^2^ for women by a bioelectrical impedance analysis. A low muscle strength was defined as a handgrip strength < 28 kg for men and <18 kg for women. Low physical performance was defined as a usual gait speed < 1.0 m/s. Sarcopenia required a low muscle mass plus a low muscle strength and/or low physical performance; severe sarcopenia required all three components [2]. The de-identified analytic file retained for this secondary report preserved the derived AWGS 2019 sarcopenia and severe-sarcopenia classifications, but not the full raw component values needed to independently regenerate the outcome for all of the participants.

Muscle mass: Muscle mass was measured using a bioelectrical impedance analysis with a multi-frequency device (InBody 770, InBody Co., Ltd., Seoul, Republic of Korea). The measurements were performed according to the manufacturer’s instructions under a standardized protocol. The participants wore light clothing; removed shoes, socks, and metallic accessories; and stood barefoot on the device electrodes. To reduce measurement variability, the participants were assessed after resting briefly, were asked to avoid vigorous physical activity before the measurement, and were assessed without an obvious acute illness or clinically apparent fluid imbalance. Participants with implanted electrical devices, limb amputation, or marked fluid retention were excluded from this assessment because these conditions could substantially affect the BIA validity. A low muscle mass was defined as an appendicular skeletal muscle mass index < 7.0 kg/m^2^ for men and <5.7 kg/m^2^ for women.

Muscle strength: Grip strength was assessed usingan InGrip handgrip dynamometer (InBody Co., Ltd., Seoul, Republic of Korea). The measurements were obtained in a standard seated position with the shoulder adducted, the elbow flexed at approximately 90 degrees, the forearm in a neutral position, and the wrist in a comfortable neutral-to-slightly extended position. Each participant performed repeated maximal voluntary contractions, and the highest valid value was retained for the analysis. A low muscle strength was defined as a handgrip strength < 28 kg for men and <18 kg for women.

Physical performance: Physical performance was evaluated by the usual gait speed using a 15-foot walk test and a handheld stopwatch. The participants were instructed to walk at their usual comfortable pace, and their gait speed was calculated in meters per second. A low physical performance was defined as a usual gait speed < 1.0 m/s.

Chronic spinal pain: The severity of chronic spinal pain was assessed using the visual analogue scale (VAS, 0–10 points). For the analysis, the pain severity was categorized as mild/moderate pain (VAS < 7) or severe pain (VAS 7–10), consistent with the categories reported in Section 3 [8]. This dichotomization was prespecified for clinical interpretability and to maintain adequate cell sizes in the small sample. The VAS was not modeled as a continuous predictor because the analysis was exploratory and the available sample was insufficient to assess nonlinear pain–response relationships reliably.

Body mass index: The BMI was initially coded as underweight (<18.5 kg/m^2^) versus non-underweight (≥18.5 kg/m^2^). The available analytic dataset did not retain separate overweight and obesity categories; therefore, sarcopenic obesity could not be evaluated. To avoid misclassification in the reporting, the former label “normal BMI” was corrected to “non-underweight BMI,” and this limitation was explicitly acknowledged.

### 2.4. AWGS 2025 Framework and Reclassification Feasibility

AWGS 2019 remained the prespecified primary classification. The AWGS 2025 framework defines sarcopenia as the concurrent presence of a low muscle mass and a low muscle strength [11]. For adults aged 60–64 years, the height-adjusted BIA thresholds are an ASMI < 7.6 kg/m^2^ for men and <5.7 kg/m^2^ for women, with a handgrip strength < 34 kg for men and <20 kg for women. For adults aged ≥ 65 years, the corresponding thresholds are an ASMI < 7.0 kg/m^2^ for men and <5.7 kg/m^2^ for women, with a handgrip strength < 28 kg for men and <18 kg for women. The gait speed was retained as an outcome measure, but was not required for the updated diagnosis. A participant-level reclassification was considered; however, the retained de-identified participant-level analytic dataset available for this secondary analysis contained one record per participant, the derived AWGS 2019 sarcopenia and severe-sarcopenia status, and the covariates used in the reported regression and CHAID analyses. It did not contain the original continuous age, the ASMI, the handgrip-strength values, or a complete raw component record sufficient to independently reconstruct the AWGS 2019 status or apply AWGS 2025 thresholds. No complete retrievable raw measurement record sufficient for full-outcome reconstruction was identified in the accessible study archive. The retained age grouping also combined ages 60–65 years, preventing the application of the separate 60–64-year thresholds. Therefore, no AWGS 2025 prevalence, cross-classification, agreement percentage, or Cohen’s κ were calculated.

### 2.5. Bias Control

Several procedures were applied to reduce potential sources of bias. Predefined eligibility criteria and a single recruitment setting were used, but convenience sampling and the incomplete screening log may have introduced selection bias. To reduce the measurement bias, sarcopenia components were assessed according to AWGS 2019 thresholds, using the same BIA device, InGrip handgrip dynamometer (InBody Co., Ltd., Seoul, Republic of Korea), and 15-foot gait-speed procedurethroughout data collection. Interview-based variables were collected using a structured questionnaire and predefined recall periods, particularly fora history of falls in the previous 12 months. Potential confounding was addressed analytically by multivariable logistic regression, including clinically relevant variables and variables showing potential bivariable associations. However, residual confounding was likely because physical activity, nutrition-related variables, rehabilitation exposure, the inflammatory burden, the pain duration, and detailed body-composition phenotypes were not comprehensively measured.

### 2.6. Statistical Analysis

All the data were double-entered using EpiData 3.1 (EpiData Association, Odense, Denmark) and analyzed using IBM SPSS Statistics for Windows, version 27.0 (IBM Corp., Armonk, NY, USA).

Descriptive statistics summarized the participant characteristics and the verified diagnostic categories (KOA only, chronic spinal pain only, and both conditions). Categorical variables are reported as n (%); continuous variables are reported as the mean ± standard deviation or median (range), as appropriate. Source-record verification showed that all of the participants belonged to the combined-condition category; consequently, no formal between-category comparisons were conducted.

Bivariable analyses explored factors associated with AWGS 2019-defined sarcopenia. Clinically relevant variables and variables showing potential bivariable associations were entered into a multivariable logistic regression model to estimate the adjusted odds ratios (AORs) and 95% CIs. The age group was coded as 60–65, 66–70, or >70 years, with 60–65 years as the reference. Low education was coded as below primary school versus primary school or higher. The living situation, occupation, fall history, and chronic spinal pain severity were categorical; the PSQI was continuous. Because 36 events supported eight coefficients, the model was considered exploratory. The results are described as adjusted associations, not independent predictors, and may be affected by sparse-data bias, residual confounding, and coefficient instability.

An AWGS 2025 cross-classification was not performed because the retained de-identified participant-level analytic dataset did not contain the original continuous age, the ASMI, the handgrip values, or the complete raw component record required to apply the updated age-specific definition jointly. The dataset did retain the derived AWGS 2019 sarcopenia status and the covariates used in the reported regression and CHAID analyses. The analyses were therefore reproducible only conditional on the retained AWGS 2019 outcome, not by the independent reconstruction of that outcome from raw measurements. The updated prevalence, directional reclassification counts, overall agreement, and Cohen’s κ were not estimated, because the retained age grouping and unavailable raw component values could not reconstruct the updated case status.

A chi-square automatic interaction detection (CHAID) tree was retained only as a descriptive, hypothesis-generating partitioning display of within-sample sarcopenia proportions. The tree was developed and viewed in the same dataset, without cross-validation, a bootstrap stability analysis, pruning validation, or external validation. No apparent classification accuracy, discrimination statistic, or clinical decision-rule utility is reported. A two-sided *p*-value < 0.05 was used for the exploratory regression analysis. No missing data were identified for the variables included in the final analysis.

### 2.7. Ethical Considerations

Written informed consent was obtained from all of the participants prior to data collection. Participant confidentiality was protected by using study codes instead of personal identifiers, restricting access to the dataset to the research team, and reporting only aggregate findings.

## 3. Results

### 3.1. Participant Characteristics and Prevalence of Sarcopenia

**Figure 1:**
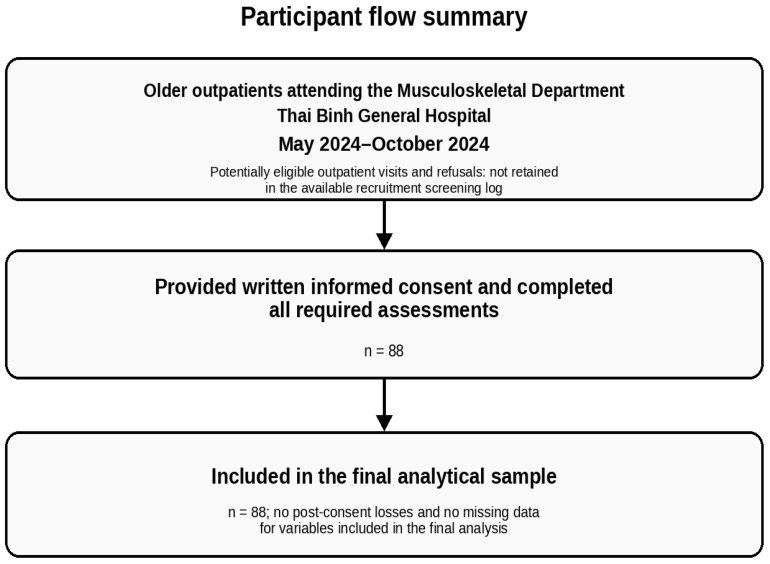
Participant flow summary for recruitment and analysis. Note: The original recruitment screening log did not retain the total number of all potentially eligible outpatient visits or refusals; therefore, the diagram reports the traceable enrollment and analysis stages available from the study records.

The retained recruitment documentation did not include a complete screening log for all potentially eligible visits or refusals. Eighty-eight participants provided written consent, completed all the required assessments, and were included in the final analysis. There were no post-consent losses and no missing values for variables used in the final secondary analyses. The retained participant-level file was complete for the derived AWGS 2019 outcome and secondary-analysis covariates, but not for the original raw AWGS component measurements needed for full outcome reconstruction (Figure 1; Table 1).

The sample included 88 participants (mean age: 70.5 ± 6.7 years), of whom 69 (78.4%) were women. The core AWGS 2019 prevalence and age association were reported in the related cohort article [10] and are repeated here to define the analytic sample for the secondary analyses. Eight participants (9.1%) met the Fried frailty phenotype. Under AWGS 2019, 36 participants (40.9%) had sarcopenia: 21 (23.9%) had sarcopenia without all three components and 15 (17.0%) had severe sarcopenia. Women represented 28/36 of the participants with sarcopenia (77.8%) and 41/52 without sarcopenia (78.8%). Frailty and sarcopenia coexisted in seven participants (7.9%). Frailty was summarized descriptively and was not entered into the regression model because only eight participants were frail and the Fried phenotype overlaps with weakness and slowness. The retained dataset preserved the derived AWGS 2019 classification, but not a complete raw measurement record that would allow for the independent reconstruction of the main outcome.

### 3.2. Diagnostic-Category Composition and Feasibility of AWGS 2025 Reclassification

After source-record verification of the retained participant list, diagnostic fields, and related report, the diagnostic composition was KOA only, 0/88 (0.0%); chronic spinal pain only, 0/88 (0.0%); and both conditions, 88/88 (100.0%). Among the participants with both conditions, 36/88 (40.9%) met the AWGS 2019 sarcopenia criteria and 52/88 (59.1%) did not. Because the two single-condition categories were empty, a between-category comparison was not applicable (Table 2).

A numerical AWGS 2025 reclassification was not performed. The retained participant-level analytic dataset included one record per participant, the derived AWGS 2019 sarcopenia and severe-sarcopenia status, and covariates used in the regression and CHAID analyses. It did not preserve the original continuous age, ASMI, handgrip-strength values, or complete raw component records sufficient to independently reconstruct the AWGS 2019 status or establish concurrent low muscle mass and low muscle strength under the age-specific AWGS 2025 thresholds. The retained 60–65-year age category also could not distinguish ages 60–64 from age 65. Consequently, the updated prevalence, reclassification counts in each direction, percentage agreement, and Cohen’s κ could not be calculated.

### 3.3. Exploratory Factors Associated with Sarcopenia

Table 3 presents the age-group and sex distribution in relation to the binary sarcopenia status. Women accounted for 20/28 of the participants aged 60–65 years (71.4%), 26/30 of the participants aged 66–70 years (86.7%), and 23/30 of the participants aged > 70 years (76.7%). The proportion of women was similar in the sarcopenia and no-sarcopenia groups (77.8% vs. 78.8%). Sarcopenia became more frequent across increasing age groups, from 17.9% in the 60–65-year group to 66.7% in the >70-year group.

Table 4 presents additional demographic, lifestyle, and BMI characteristics according to the sarcopenia status. A higher proportion of farmers was observed in the sarcopenia group than in the non-sarcopenia group (83.3% vs. 61.5%, *p* < 0.05). Other variables, including the living situation, BMI category, smoking, and alcohol consumption, were not significantly associated with sarcopenia in the bivariable analysis.

With respect to clinical and pathological characteristics (Table 5), a history of falls was associated with sarcopenia. Among the participants with sarcopenia, 36.1% reported a history of falls, compared with 11.5% among those without sarcopenia (*p* < 0.001). The severity of knee osteoarthritis and chronic spinal pain did not differ significantly between the two groups in the bivariable comparison.

**Figure 2:**
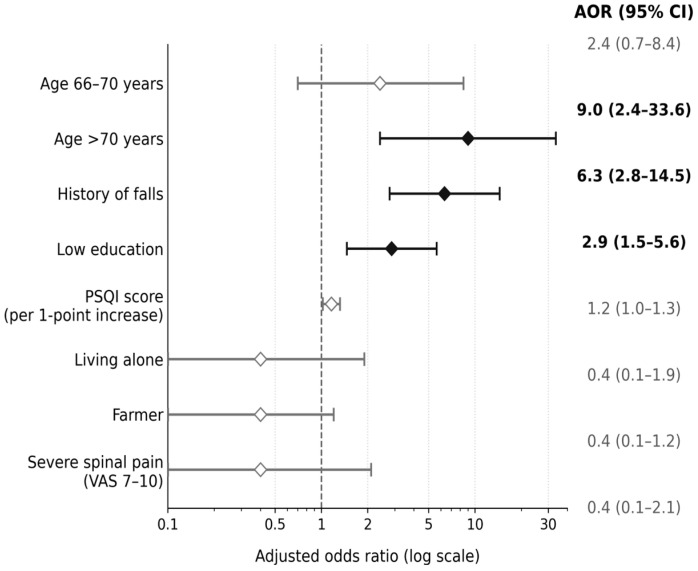
Forest plot of adjusted odds ratios for factors associated with sarcopenia in the exploratory multivariable logistic regression model.

**Figure 3:**
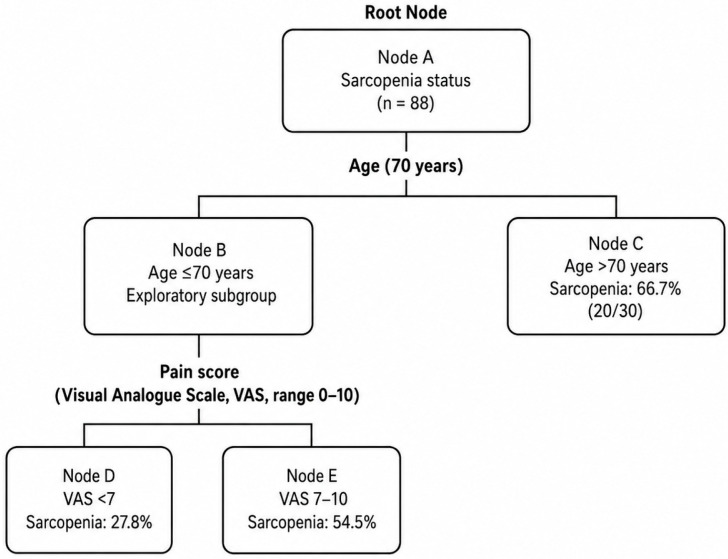
Descriptive, hypothesis-generating CHAID partitioning display for sarcopenia status.

The results of the multivariate logistic regression analysis of factors associated with muscle atrophy (Table 6) showed that age > 70 years (AOR = 9.00; 95% CI = 2.40–33.60), history of falls (AOR = 6.33; 95% CI = 2.77–14.45), low educational attainment (AOR = 2.86; 95% CI = 1.46–5.61), and higher PSQI scores (AOR = 1.16 per point; 95% CI = 1.02–1.32) were associated with adjusted muscle atrophy.after adjustment. The age estimate was also reported in the related article [10]; the fall history, education, and PSQI results are presented here as additional exploratory adjusted associations. Wide CIs, particularly for age and falls, indicate substantial imprecision and possible coefficient instability. Occupation, living situation, and chronic spinal pain severity did not remain statistically significant after adjustment (Figure 2 and Figure 3).

In this sample, age group formed the first CHAID split. Among the participants aged ≤ 70 years, chronic spinal pain severity formed a subsequent split, with sarcopenia proportions of 54.5% for severe pain and 27.8% for mild/moderate pain. This display was derived and viewed in the same dataset without resampling or validation. It is presented only to generate hypotheses about possible subgroup patterns and does not demonstrate the predictive performance, stability, or clinical decision-rule utility.

## 4. Discussion

The core AWGS 2019 sarcopenia proportion and age association from this 88-participant sample were previously reported in a related publication [10]. The present manuscript should therefore be read as a secondary exploratory analysis of a previously reported or substantially overlapping cohort, not as an independent cohort study or independent replication. Its incremental contribution is limited, but specific: it clarifies that all participants had coexisting KOA and chronic spinal pain after source-record verification; evaluates additional routinely collected associations involving the fall history, educational attainment, sleep quality, and pain severity; presents a sample-specific CHAID display; and interprets the AWGS 2019 estimate in light of AWGS 2025. These elements remain descriptive and hypothesis-generating, are conditional on the retained derived AWGS 2019 outcome, and do not establish a new predictive model or definitive clinical risk-stratification tool.

The AWGS 2025 update remains important for interpreting the 40.9% AWGS 2019 estimate. AWGS 2025 requires a low muscle mass and a low muscle strength concurrently, removes gait speed from the diagnostic requirement, and applies higher age-specific thresholds to participants aged 60–64 years [11]. Because the retained participant-level analytic dataset did not preserve the original continuous age, ASMI, grip-strength measurements, or complete raw component records, the direction and magnitude of reclassification could not be quantified. No complete retrievable raw measurement records sufficient for full outcome reconstruction were identified in the accessible study archive, so the original AWGS 2019 outcome could not be independently regenerated during this secondary revision. The retained 60–65-year age category also prevented the separate application of the 60–64-year thresholds. The AWGS 2019 proportion should therefore not be compared directly with estimates derived under AWGS 2025.

An age > 70 years showed the largest adjusted association with AWGS 2019-defined sarcopenia. This is biologically plausible given age-related declines in muscle mass, neuromuscular reserve, anabolic responsiveness, and physical function [2,3]. However, the magnitude should not be overinterpreted: the CI was wide, the events-per-coefficient ratio was low, and the cross-sectional design cannot establish temporality.

Fall history also remained associated with sarcopenia after adjustment. The relationship may be bidirectional: an impaired strength and performance can increase instability, while falls may lead to a fear of movement, lower activity, and deconditioning [3]. Because physical activity, rehabilitation exposure, and environmental fall hazards were not measured, fall history should be viewed as a pragmatic case-finding cue that warrants further assessment, not as a causal predictor.

Low educational attainment and poorer sleep quality also remained associated in the exploratory model. These findings may reflect broader social and behavioral pathways, including health literacy, access to exercise and nutrition counseling, pain perception, and rehabilitation adherence. Because nutrition, protein intake, malnutrition risk, and habitual physical activity were not measured, these results are adjusted correlations rather than evidence of independent causal effects.

The chronic spinal pain severity was not statistically significant in the adjusted logistic model, but appeared in a later split of the descriptive CHAID display among participants aged ≤70 years. This discordance should not be treated as evidence of a validated interaction. Pain-related activity avoidance and disuse are plausible hypotheses, but physical activity, kinesiophobia, inflammatory biomarkers, and pain duration were not measured [12,13]. The CHAID pattern therefore serves only to motivate adequately powered confirmatory studies.

The coexistence of frailty and sarcopenia observed in our sample is also clinically plausible, as previous studies have shown that these two geriatric syndromes frequently overlap in older medical patients [14]. Frailty was not included in the primary regression model because only eight participants were frail and because the Fried phenotype includes weakness and slowness, which overlap directly with sarcopenia components. This analytic decision reduces the risk of sparse-data bias and overadjustment, but it also limits the ability to distinguish whether frailty acts as a confounder, mediator, or coexisting syndrome. Future studies should measure frailty, sarcopenia, disability, and physical activity using a prespecified conceptual framework and larger samples.

The new clinical meaning of this report is modest and should be stated precisely. It provides a local benchmark for a selected musculoskeletal outpatient service, identifies readily obtainable characteristics that may justify confirmatory muscle assessments, clarifies that the cohort consisted entirely of patients with coexisting KOA and chronic spinal pain, and specifies the participant-level measurements that should be retained for future AWGS 2025 reclassification and auditability. These contributions are descriptive and hypothesis-generating; they do not validate a screening score, prediction algorithm, or treatment pathway.

Musculoskeletal outpatient clinics may be a feasible point of contact for sarcopenia case-finding, particularly when older patients report falls or functional decline. Nevertheless, the present associations cannot be converted into a formal score or decision rule. Any targeted case-finding strategy requires prospective validation in larger, multicenter samples and should incorporate physical activity, the nutritional status, the rehabilitation history, and a broader geriatric assessment.

The external validity is limited because the sample came from one musculoskeletal outpatient department in Northern Vietnam and was predominantly female, with a high proportion of farmers and a selected burden of chronic musculoskeletal disease. The results should not be generalized to community-dwelling adults, other outpatient specialties, broader geriatric populations, or patients with isolated KOA or isolated chronic spinal pain without confirmation.

Several limitations require emphasis. First, the cross-sectional design precludes causal and temporal inference. Second, the sample was modest for a multivariable analysis: 36 events supported eight coefficients, raising the possibility of sparse-data bias, unstable coefficients, and inflated odds ratios. Third, the cohort was previously described in a related publication, and exact participant-level overlap could not be reconstructed because a linkage key was not retained; this report must therefore not be interpreted as an independent replication. Fourth, convenience sampling from a single center, the incomplete screening log, the underrepresentation of men, and the absence of KOA-only and chronic-spinal-pain-only comparison groups restrict the generalizability and preclude diagnostic-subgroup comparisons. Fifth, habitual physical activity, dietary and protein intake, malnutrition risk, rehabilitation exposure, inflammatory biomarkers, pain duration, and detailed body-composition phenotypes were not measured, leaving substantial residual confounding. Sixth, the BMI was retained only as underweight versus non-underweight, so obesity and sarcopenic obesity could not be assessed. Seventh, the retained dataset preserved the derived AWGS 2019 sarcopenia status and secondary-analysis covariates, but not the raw component measurements needed to independently reconstruct the AWGS 2019 status; this limits dataset auditability. Eighth, AWGS 2025 reclassification could not be performed because the retained participant-level analytic dataset lacked the original continuous age, ASMI, handgrip values, and complete component records needed to apply the updated age-specific definition. Finally, the CHAID display was not internally or externally validated and has no demonstrated predictive or decision-rule utility.

Within these constraints, this secondary manuscript offers local descriptive evidence, transparent reporting of its relationship to the prior publication, confirmation that all of the participants had both musculoskeletal conditions, and explicit acknowledgment that AWGS 2019 and AWGS 2025 estimates are not directly interchangeable. Its auditability is limited to the reproduction of the secondary analyses conditional on the retained derived AWGS 2019 outcome, not the independent reconstruction of the original sarcopenia classification. Larger, multicenter longitudinal studies with a balanced sex representation, prespecified muscle-health frameworks, retained participant-level muscle measurements, fuller measurements of nutrition and activity, and formal model validation are needed.

## 5. Conclusions

In this secondary analysis of a previously described or substantially overlapping cohort, a substantial proportion of the selected Vietnamese musculoskeletal outpatient sample met AWGS 2019 sarcopenia criteria. The core prevalence and age association should not be interpreted as independent replication. Source-record verification confirmed that all of the participants had coexisting KOA and chronic spinal pain, so a diagnostic-subgroup comparison was not possible and the findings should not be generalized to isolated-condition populations. AWGS 2025 reclassification could not be completed because the retained participant-level analytic dataset lacked the original continuous age, ASMI, handgrip values, and complete raw component records needed for the updated age-specific definition; accordingly, the AWGS 2019 estimate should not be treated as directly interchangeable with estimates generated under the updated framework. An older age, the fall history, low educational attainment, and poorer sleep quality remained associated with sarcopenia in an imprecise exploratory model, while the CHAID display showed only within-sample hypothesis-generating patterns. The report’s incremental contribution is local, descriptive, and hypothesis-generating; it is not a fully auditable reconstruction of the original AWGS measurements and should not be used as a validated risk score or clinical decision rule.

## Figures and Tables

**Table 1:** Frailty and binary sarcopenia status among participants, with female distribution (n = 88).

Characteristics	n	%
Frailty status (Fried criteria)		
Frail	8	9.1
Non-frail	80	90.9
**Sarcopenia status (AWGS 2019)**		
No sarcopenia	52	59.1
Sarcopenia	21	23.9
Severe sarcopenia	15	17.0
Coexistence of frailty and sarcopenia	7	7.9

**Table 2:** Diagnostic categories and AWGS 2019 sarcopenia status (n = 88).

Diagnostic Category	Total, n (%)	Sarcopenia, n (% Within Category)	No Sarcopenia, n (% Within Category)
KOA only	0 (0.0%)	Not applicable	Not applicable
Chronic spinal pain only	0 (0.0%)	Not applicable	Not applicable
Both conditions	88 (100.0%)	36 (40.9%)	52 (59.1%)
Total	88 (100.0%)	36 (40.9%)	52 (59.1%)

**Table 3:** Age-group and sex distribution according to binary sarcopenia status (n = 88).

Characteristic	Total, n (%)	Female, n (% Within Row)	Male, n (% Within Row)	Sarcopenia, n (% Within Row)	No Sarcopenia, n (% Within Row)
Age group					
60–65 years	28 (31.8)	20 (71.4)	8 (28.6)	5 (17.9)	23 (82.1)
66–70 years	30 (34.1)	26 (86.7)	4 (13.3)	11 (36.7)	19 (63.3)
>70 years	30 (34.1)	23 (76.7)	7 (23.3)	20 (66.7)	10 (33.3)
Sarcopenia status					
No sarcopenia	52 (59.1)	41 (78.8)	11 (21.2)	-	-
Sarcopenia, including severe sarcopenia	36 (40.9)	28 (77.8)	8 (22.2)	-	-
Total	88 (100.0)	69 (78.4)	19 (21.6)	36 (40.9)	52 (59.1)

Note: The percentages in the female and male columns were calculated within each age group or sarcopenia-status row. The percentages in the sarcopenia and no-sarcopenia columns were calculated within each age group.

**Table 4:** Additional demographic, lifestyle, and BMI characteristics according to sarcopenia status (n = 88).

Characteristics	Sarcopenia (n = 36) n (%)	No Sarcopenia (n = 52) n (%)	*p*-Value
Occupation			<0.05
Farmer	30 (83.3)	32 (61.5)	
Others (civil servant, freelance, etc.)	6 (16.7)	20 (38.5)	
Living situation			Not significant
Living alone	6 (16.7)	5 (9.6)	
Living with relatives	30 (83.3)	47 (90.4)	
Body mass index			Not significant
Underweight (<18.5 kg/m^2^)	8 (22.2)	13 (25.0)	
Non-underweight (≥18.5 kg/m^2^) *	28 (77.8)	39 (75.0)	
Lifestyle habits			
Smoking (yes)	3 (8.3)	4 (7.7)	Not significant
Alcohol consumption (yes)	7 (19.4)	9 (17.3)	Not significant

* The BMI category was corrected from “normal” to “non-underweight” because the available analytic dataset did not retain separate overweight and obesity categories.

**Table 5:** Clinical and pathological characteristics according to sarcopenia status (n = 88).

Clinical and Pathological Characteristics	Sarcopenia (n = 36) n (%)	No Sarcopenia (n = 52) n (%)	*p*-Value
Hypertension			Not significant
Yes	9 (25.0)	17 (32.7)	
No	27 (75.0)	35 (67.3)	
Diabetes mellitus			Not significant
Yes	3 (8.3)	8 (15.4)	
No	33 (91.7)	44 (84.6)	
Chronic kidney disease			Not significant
Yes	3 (8.3)	8 (15.4)	
No	33 (91.7)	44 (84.6)	
Severity of knee osteoarthritis			Not significant
Grade 3–4 (severe)	28 (77.8)	38 (73.1)	
Grade 1–2 (mild/moderate)	8 (22.2)	14 (26.9)	
Chronic spinal pain severity			Not significant
Severe pain (VAS 7–10)	27 (75.0)	39 (75.0)	
Mild/moderate pain (VAS < 7)	9 (25.0)	13 (25.0)	
History of falls			<0.001
Yes	13 (36.1)	6 (11.5)	
No	23 (63.9)	46 (88.5)	

**Table 6:** Multivariable logistic regression analysis of factors associated with sarcopenia (n = 88).

Variables	Category	AOR	95% CI	*p*-Value
Age group	60–65 years (Ref.)	1.00	-	-
	66–70 years	2.4	0.7–8.4	Not significant
	>70 years	9.00	2.40–33.60	<0.01
History of falls	No (Ref.)	1.00	-	-
	Yes	6.33	2.77–14.45	<0.001
Low education (under primary school)	No (Ref.)	1.00	-	-
	Yes	2.86	1.46–5.61	<0.01
PSQI score				
	Per 1-point increase	1.16	1.02–1.32	0.03
Living situation	With family (Ref.)	1.00	-	-
	Living alone	0.4	0.1–1.9	Not significant
Occupation	Other occupation (Ref.)	1.00	-	-
	Farmer	0.4	0.1–1.2	Not significant
Chronic spinal pain severity	Mild/moderate pain (VAS < 7) (Ref.)	1.00	-	-
	Severe pain (VAS 7–10)	0.4	0.1–2.1	Not significant

AOR: adjusted odds ratio; CI: confidence interval; PSQI: Pittsburgh Sleep Quality Index. Estimates are exploratory adjusted associations.

## Data Availability

The de-identified participant-level analytic dataset retained for this secondary analysis contains one record for each of the 88 participants, the derived AWGS 2019 sarcopenia and severe-sarcopenia classifications, and the demographic and clinical covariates reported in the manuscript and used in the descriptive, logistic-regression, and CHAID analyses. It is available from the corresponding author upon reasonable request, subject to applicable ethical and privacy restrictions. The dataset does not contain the original continuous age, ASMI, handgrip-strength values, or complete participant-level set of raw component measurements sufficient to independently reconstruct AWGS 2019 classifications. No complete retrievable raw measurement records sufficient for full outcome reconstruction were identified in the study archive accessible for this revision. Accordingly, the shared dataset permits the reproduction of the reported secondary analyses conditional on the retained AWGS 2019 outcome, but not independent reconstruction of the AWGS 2019 status or reclassification under AWGS 2025.

## Abbreviations

The following abbreviations are used in this manuscript:

AOR	Adjusted odds ratio
ASMI	Appendicular skeletal muscle mass index
AWGS	Asian Working Group for Sarcopenia
BIA	Bioelectrical impedance analysis
BMI	Body mass index
CHAID	Chi-square automatic interaction detection
CI	Confidence interval
KOA	Knee osteoarthritis
PSQI	Pittsburgh Sleep Quality Index
SD	Standard deviation
SE	Standard error
VAS	Visual analogue scale
